# Monitoring Epoxy Coated Steel under Combined Mechanical Loads and Corrosion Using Fiber Bragg Grating Sensors

**DOI:** 10.3390/s22208034

**Published:** 2022-10-21

**Authors:** Luyang Xu, Dawei Zhang, Ying Huang, Shuomang Shi, Hong Pan, Yi Bao

**Affiliations:** 1 Department of Civil, Construction, and Environmental Engineering, North Dakota State University, Fargo, ND 58102, USA; 2 Department of Civil, Environmental and Ocean Engineering, Stevens Institute of Technology, Hoboken, NJ 07030, USA

**Keywords:** corrosion, fiber Bragg grating (FBG) sensors, interactive anomalies, structural health monitoring (SHM)

## Abstract

Fiber Bragg grating (FBG) sensors have been applied to assess strains, stresses, loads, corrosion, and temperature for structural health monitoring (SHM) of steel infrastructure, such as buildings, bridges, and pipelines. Since a single FBG sensor measures a particular parameter at a local spot, it is challenging to detect different types of anomalies and interactions of anomalies. This paper presents an approach to assess interactive anomalies caused by mechanical loading and corrosion on epoxy coated steel substrates using FBG sensors in real time. Experiments were performed by comparing the monitored center wavelength changes in the conditions with loading only, corrosion only, and simultaneous loading and corrosion. The theoretical and experimental results indicated that there were significant interactive influences between loading and corrosion for steel substrates. Loading accelerated the progress of corrosion for the epoxy coated steel substrate, especially when delamination in the epoxy coating was noticed. Through the real-time monitoring from the FBG sensors, the interactions between the anomalies induced by the loading and corrosion can be quantitatively evaluated through the corrosion depth and the loading contact length. These fundamental understandings of the interactions of different anomalies on steel structures can provide valuable information to engineers for better management of steel structures.

## 1. Introduction

Reinforced concrete (RC) and steel are the most common construction materials for civil infrastructures [1,2]. As time goes, civil infrastructures made by reinforced concrete or steel will deteriorate and anomalies or damages may accumulate in the structures, which may result in safety concerns for structures that lead to tragedies of property and life losses. Among the various types of structural degradations, steel corrosion and local stress accumulations are well known as some of the main concerns/threats to the structural integrity, durability, and reliability of RC and steel structures. To prevent and mitigate steel corrosion, various protective measures can be applied to steel in the RC or steel structures, such as cathodic protection, corrosion inhibitor, anti-corrosion alloys, and coatings. Cathodic protection mitigates corrosion through the use of the metal as the cathode of an electrochemical cell [3,4]. Corrosion inhibitor prevents access of the corrosive substance to the metal, thus, prevents corrosion [5,6]. The anti-corrosion alloys form corrosion-resistant alloys, such as stainless steel alloys and aluminum alloys, through adding high corrosion-resistant components into the steel materials [7]. Compared to the above-mentioned corrosion mitigation approaches, the application of anti-corrosive coatings is a cost-effective method to protect metals with additional benefit to reduce local stress accumulations since they provide an extra layer between the substrates and loading [8,9]. Among the anti-corrosive coatings, a thin layer of epoxy coating is one of the most commonly used coatings to isolate structures from the external corrosive environment for steel corrosion protection [10,11,12].

In addition to protective coatings, to detect steel corrosion and loading-induced damages, in practice, visual inspection is commonly applied due to its simplicity. However, the visual inspection may not be accurate, as the occurrence of corrosion or loading-induced damages are usually located beneath the protective coatings and are hard to see directly, and it may not be timely due to the large scale of the associated structures [13]. Searching for a more reliable inspection method for steel corrosion, weight loss coupon measurement is widely used in practice, in which steel coupons are placed at the location of concerns and weight losses are measured after a period time of exposure to estimate corrosion rates [14]. This approach is more quantitive compared to visual inspection, however, it suffers from some unavoidable drawbacks, such as long inspection time, high maintenance, and labor costs. Additionally, this method cannot detect loading-induced damages on the structure and also may require disassembly of a part of structures resulting in a loss of structural integrity.

To address the limitations of the above-mentioned traditional approaches to detect steel corrosion and local stress accumulations, non-destructive evaluation (NDE) methods have become popular. The NDE methods do not bring any interruptions or damages to the original structures, and also provide high accuracy and ease of use. However, most NDE techniques are highly dependent on the applied sensors which mostly rely on the transmission and detection of electrical signals, such as acoustic and ultrasonic sensors, magnetic sensors, microwave, or guided wave sensors, etc., resulting in lack of detection accuracy with the existence of electrical–magnetic interference in the field applications [15,16]. More importantly, as the NDE techniques are mostly external to a structure, their application may require additional tool sets and skilled labor. Therefore, the NDE techniques are usually used for scheduled periodic inspections or needed inspections and may not be cost effective for wide and long-term monitoring applications [2].

In recent years, structural health monitoring (SHM) has been widely recognized as an effective onsite monitoring method of early abnormalities in many industries, such as aerospace, mechanical, and civil engineering fields [17,18]. A proper SHM system can detect early initiation of degradations and monitor the cumulation of degradations and damages, such as overstress, cracks, and corrosion in existing and new structures to guide a cost-effective structural maintenance plan and avoid collapses and the resultant tragedies [19,20]. There are many types of sensors can be used in an SHM system, such as electrical strain gauges, acoustic and ultrasonic sensors, cameras, and fiber optic sensors [21,22,23]. Among the various existing sensors for SHM systems, fiber optic sensors, especially fiber Bragg grating (FBG) sensors, attract intensive attention from researchers due to the unique advantages of high sensitivity and precision, satisfiable linearity, immunity to electromagnetic interference, resistance to harsh environments, light weight, small physical dimensions, easy-operability, and relatively low cost [2,24]. The FBG sensors, as the most commonly used fiber optic sensors, have been applied in many industries for the monitoring of strains, stress, loads, and temperatures [17]. As corrosion can be investigated by monitoring strain changes inside or on the surface of a steel structural component, FBG sensors have also been applied to detect steel corrosion for bare and coated steel structures [25,26,27].

Although FBG sensors have been used as a tool to monitor structural performance and damages, as an FBG sensor belongs to a category of local point sensors, most of the research used FBG sensors to detect one single measure of structural performance or damage. However, structures may not only suffer from corrosion, but also have damages induced by accumulation of local stresses as various natural factors, such as wind, snow, and moving loads may introduce extreme loads to structures. 

Recent investigations have shown that the interaction of loading and corrosion could mutually promote damages induced by each other, which may pose threats to the health condition and safety of structures. Accelerated corrosion results from previous research [28] indicated that the compressive load level could simultaneously influence the corrosion rate of a steel bar and the corrosive cracking behavior of reinforced concrete. Through the evaluation of the deflections of RC beams under load and accelerated corrosion, it was noticed that during the initial stage of corrosion, the deflections of beams increased significantly due to the flexural tension and the expansive stresses caused by corrosion products, resulting in crack propagations on the tension sides of beams [29]. In addition, researchers found that both the corrosion of reinforcements and the cyclic loading obviously reduced the bond properties of concrete structures [30]. Severe corrosion led to significant degradation of the bond during the first five cycles, the corrosion process was weakened with loading, and the corrosion of rebars under compression was more severe than that under tension [31,32].

However, to date, there is yet to be real-time monitoring of the interaction between corrosion and load-induced damages on steel structures. In this paper, systematic experimental studies were performed to investigate such interactive influences between steel corrosion and static loads on epoxy coated steel using FBG sensors. Periodic temporary static loads were applied every six days on the coated steel substrates which were immersed in 3.5% NaCl solutions for accelerated corrosion. The interactions between the loads and corrosion were investigated by data mining the patterns of the recorded center wavelength changes of the FBG sensors.

## 2. Theoretic Analysis

### 2.1. Sensing Principles

A typical single-mode optical fiber is composed of a fused silica core and cladding. The refractive index of the core is higher than that of the cladding, resulting in total internal reflection of light waves at the core-cladding interface. The total internal reflection allows light waves to propagate along the fiber length. An FBG sensor is manufactured by engraving periodic Bragg gratings in an optical fiber. When the incident light passes through the gratings, light waves with a specific Bragg wavelength ($\lambda_{B}$) are reflected, while the rest of light waves are transmitted. The value of the Bragg wavelength ($\lambda_{B}$) of an FBG sensor can be determined using the effective refractive index ($n_{eff}$) of the fiber and the Bragg grating period (Λ) [24,33,34] as:

(1) $$\lambda_{B}=2n_{eff}\cdot \Lambda$$

The effective refractive index of a particular FBG sensor is a constant, while the fiber Bragg grating period is sensitive to temperature and strain changes [35]:

(2) $$\Delta \lambda =\lambda_{B}[\left(1-P_{e}\right)\cdot \varepsilon_{c}+\left(\alpha +\xi \right)\Delta T]$$

where Δλ is the Bragg wavelength shift; *P_e_* is the photo elastic coefficient of the fiber; $\varepsilon_{c}$ is the strain change along the grating direction; α and ξ are the thermo expansion coefficient and the thermo-optic coefficient of the fiber, respectively; and ΔT is the change of external temperature. If the temperature is unchanged or compensated using an FBG sensor that is nearby and free of strain change, Equation (2) can be rewritten as:

(3) $$\Delta \lambda =\lambda_{B}\left(1-P_{e}\right)\cdot \varepsilon_{c}$$

Strain changes along the grating can be determined by measuring the wavelength shift of a FBG sensor. The strain changes can be induced by mechanical loads, corrosion, and other effects.

### 2.2. Calibration of FBG Sensors

In this study, the FBG sensors were attached to steel specimens using a two-part epoxy. The epoxy served as adhesive and provided protection for the optical fiber. The strain sensitivity of the FBG sensor embedded in epoxy was calibrated through tension tests to eliminate the strain transfer effect [36,37,38] on the strain measurement results. The strain transfer ratio (k) is expressed as [39]:

(4) $$k=\frac{\varepsilon_{c}}{\varepsilon_{1}}$$

where *ε*_1_ is the strain in the steel plate and *ε_c_* is the strain measured from the FBG sensor.

**Figure 1:**
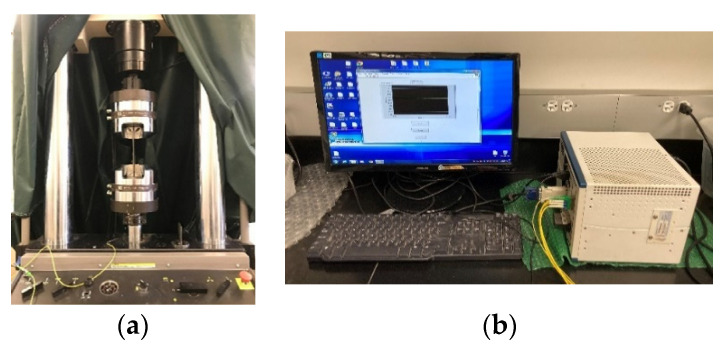
Calibration of strain transfer ratio: (**a**) tensile test setup and (**b**) FBG interrogator.

The test was performed using a Mechanical Testing and Simulation (MTS) loading machine under tension as shown in Figure 1a. The strain in the steel plate was evaluated using an extensometer, and the strain in the FBG sensor was simultaneously measured using an interrogator (model: National Instrument NI PXIe-1071) as shown in Figure 1b. The sampling rate of the interrogator and MST machine were 10 Hz. Three calibration tests were performed on the FBG strain sensor. In addition, another FBG sensor was attached to a plate which did not load tension for temperature compensation. All the calibration tests were performed at room temperature of 22 °C.

**Figure 2:**
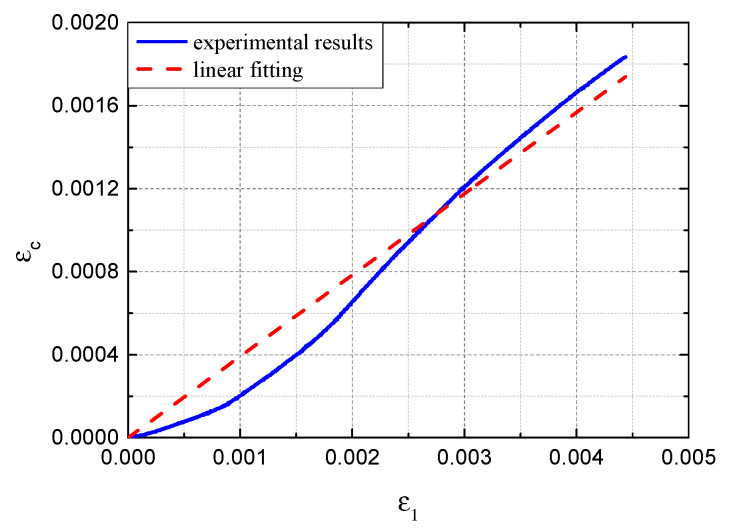
Calibration results of the strain transfer ratio of the adopted FBG sensors.

Figure 2 shows the average tensile test data after temperature compensation from the three calibration tests. The strains in the steel plate and the FBG sensor were approximately proportional. The slope of the curve represents the strain transfer ratio, which is 0.39 according to linear regression. The coefficient of determination of the linear regression analysis was 0.99. Based on Equations (3) and (4), the strain changes of epoxy-coated steel plates can be estimated by monitoring the Bragg wavelength changes under corrosion and mechanical load.

### 2.3. Measuring the Depth of Pitted Corrosion

**Figure 3:**
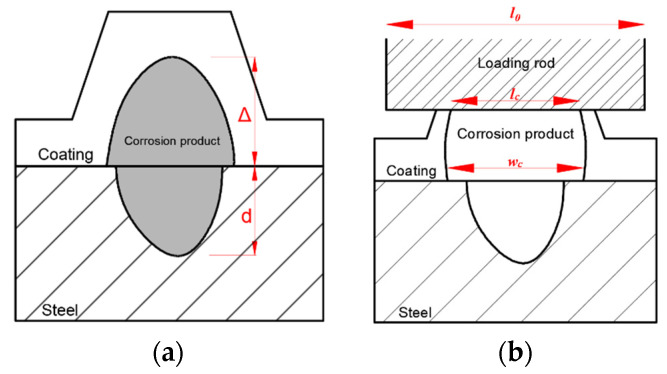
Schematic illustration of pitted corrosion effect on the deformation of the epoxy coating (**a**) deformation under only corrosion, and (**b**) deformation under loading and corrosion.

Corrosion of a local spot where the protective coating of steel is damaged is categorized as pitted corrosion. Previous studies [40,41,42,43,44,45,46] assumed that the corrosion products of pitted corrosion mainly accumulate within a small area in the vertical direction. The shape of pitted corrosion is considered as the hemispherical hole, as shown in Figure 3a. The depth of pitted corrosion (d) can be used to characterize the severity of corrosion [47]:

(5) $$d=\frac{1.2\Delta }{\left(c-1\right)}$$

where Δ is the upward displacement of coating induced by pitted corrosion, and c is the volume ratio between the corrosion product and the original steel [47].

**Figure 4:**
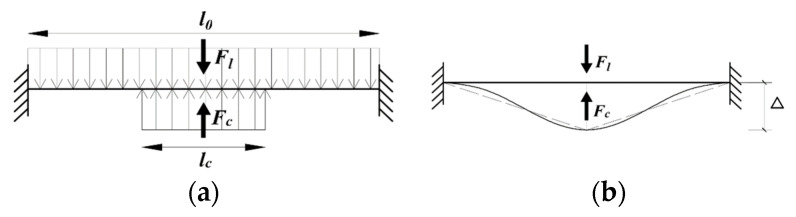
Illustration of the beam model used to mimic the combined effects of corrosion and external load on the epoxy instrumented with a FBG sensor: (**a**) illustration of the beam model and (**b**) illustration of the deformation.

If a mechanical load is applied to the location where corrosion occurs, the epoxy on top of the corrosion will be subjected to two types of forces, with one caused by the mechanical load and the other one induced by the expansion from the corrosion products, which can be simplified as concentrated loads ($F_{1}$) and ($F_{c}$) as shown in Figure 4a, respectively. As the corrosion products lift a small area of epoxy, debonding of epoxy coating would occur along the width of the corrosion. Since all other areas other than the corrosion width remain bonded to the substrate [47], the debonded epoxy can be simplified as a fixed beam, which is subjected to both uplifting force induced by corrosion products ($F_{c}$) and downward external mechanical load ($F_{1}$) as shown in Figure 4a. In other words, the FBG sensor measures the strains in the epoxy that is subjected to a combination of mechanical loads and corrosion effect.

In practical application of steel structures, comparing the large span of steel components, the deformation induced from external loads or corrosion is considered small. Thus, the dimension along steel surfaces generally remains curveless, from which it is assumed that in the deformation of the epoxy with FBG sensor under mechanical loading and corrosion can be simplified to be a triangular shape as shown in Figure 4b. When there is only external load, it can be assumed that the original length of the fixed beam model, $L_{0}$, is the diameter of the external load. The displacement at the middle span is expressed as:

(6) $$\Delta =\frac{1}{2}\sqrt{L^{2}-L_{0}^{2}}$$

where $L_{0}$ is the original length of the beam model, and L is the deformed length of the beam model.

The strains in the embedded FBG sensor are expressed as:

(7) $$\frac{L-L_{0}}{L_{0}}=\varepsilon_{c}$$

Substituting Equation (7) into Equation (6), the maximum deformation of the epoxy can be expressed as:

(8) $$\Delta =\frac{1}{2}L_{0}\cdot \sqrt{\varepsilon_{c}\cdot \left(\varepsilon_{c}+2\right)}$$

With Equations (3), (5) and (8), the depth of pitted corrosion (*d*) can be determined based on the wavelength shift of the FBG sensor:

(9) $$d=\frac{0.6L_{0}}{\left(c-1\right)}\cdot \sqrt{\alpha \Delta \lambda \cdot \left(\alpha \Delta \lambda +2\right)}$$

where $\alpha =\frac{1}{\lambda_{B}\left(1-P_{e}\right)}$.

### 2.4. Measuring the Width of Pitted Corrosion

With the progress of corrosion and the vertical accumulation of pitted corrosion products, the FBG sensor is gradually lifted. Meanwhile, with the external load on top of the corrosion products, the debonded length of the epoxy coating, which is defined as the width of pitted corrosion, $w_{c}$, continuously changes. Before extensive corrosion and under the vertical external load, the corrosion product is compacted into a thin film. Thus, the contact length of the external load and epoxy coating, $L_{0}$, can be considered equal to the initial debonded length, $w_{c}$, as shown in Figure 3b. As seen in Figure 3b, a larger width of pitted corrosion indicates a larger corrosion product diffusion area and a larger corrosion area. Thus, the width of pitted corrosion can be used to evaluate the corrosion severity and the interaction between external load and corrosion. Based on the fixed end beam model, the maximum deflection of the beam can be more accurately estimated as:

(10) $$\Delta =\frac{Fw_{c}^{3}}{384EI}$$

where $F=F_{l}-F_{c}$, E is the elastic modulus, and I is the moment of inertia.

Integrating Equations (10) and (3) into Equation (8), the relationship between the Bragg wavelength changes and the loading contact length is expressed as:

(11) $$\frac{1}{2}L_{0}\cdot \sqrt{\alpha \Delta \lambda \cdot \left(\alpha \Delta \lambda +2\right)}=\frac{Fw_{c}^{3}}{384EI}$$

where $\alpha =\frac{1}{\lambda_{B}\left(1-P_{e}\right)}$. Based on the loading only condition, by monitoring the Bragg wavelength changes under different conditions, the corresponding width of corrosion, $w_{c}$, can be obtained:

(12) $$\frac{w_{c}^{3}}{L_{0}^{3}}=\frac{\sqrt{\alpha \Delta \lambda \cdot \left(\alpha \Delta \lambda +2\right)}}{\sqrt{\alpha \Delta \lambda_{0}\cdot \left(\alpha \Delta \lambda_{l}+2\right)}}$$

where $\Delta \lambda_{0}$ is the Bragg wavelength change under the loading only. If $F_{1}\gg F_{c}$, it can be assumed that $F=F_{l}-F_{c}\approx F_{l}$. So, the width of the corrosion can be approximated as:

(13) $$w_{c}=\sqrt[{6}]{\frac{\Delta \lambda \cdot \left(\alpha \Delta \lambda +2\right)}{\Delta \lambda_{l}\cdot \left(\alpha \Delta \lambda_{l}+2\right)}}\cdot L_{0}.$$

Therefore, by monitoring the Bragg wavelength changes, both the corrosion depth (d) and the width ($w_{c}$) can be measured to investigate the interaction between damages induced by external temporary loading and corrosion.

## 3. Experimental Program

To test the interaction between steel corrosion and static load-induced damages, corrosion and periodic static loads need to be applied simultaneously. However, before applying simultaneous corrosion and loads, separate loading and corrosion tests are also needed to understand how the coated steel behaves under each of the conditions. Thus, one control test of loading only, and one control test of corrosion only was first conducted to compare with multiple tests with simultaneous loading and corrosion to investigate the interaction of loading and corrosion.

### 3.1. Materials and Dimensions

**Figure 5:**
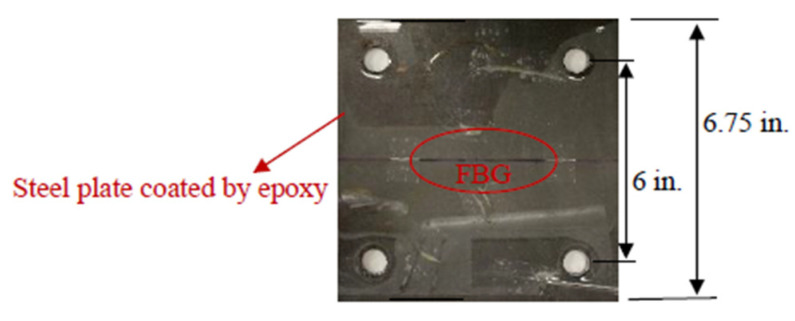
An example embedded FBG sensor.

In all the experiments, epoxy coated steel plates were used as the testing substrate. FBG sensors were embedded inside the epoxy coating at the central axis of the steel plate as shown in Figure 5. A36 structural steel was used as the material of steel plates and their dimensions were 6.75 in. × 6.75 in. × 0.125 in. Epoxy resin (Duralco 4461) was used as the protective coating. The FBG sensors (os1100) manufactured by LUNA were used and the properties of the used FBG sensors are listed in Table 1 (provided by manufacture). The same FBG interrogator as in Figure 1b was used to collect the Bragg wavelength changes.

### 3.2. Loading-Only Tests

#### 3.2.1. Test Set-Up

**Figure 6:**
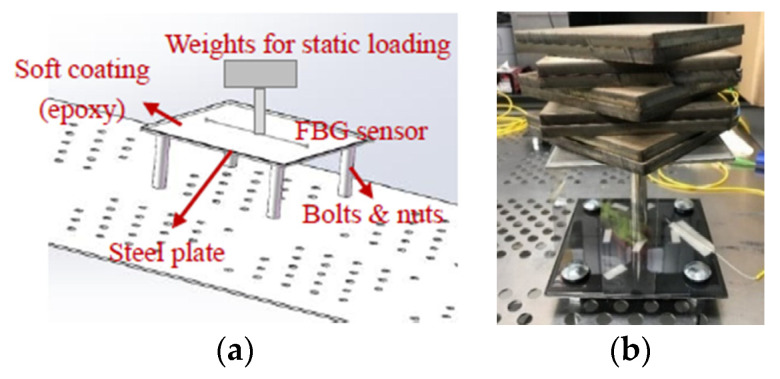
Test set-up of loading experiment: (**a**) schematic view and (**b**) a photo.

In this paper, concentrated load was applied on top of the embedded FBG sensor to investigate the loading effects. To create a loading mechanics, the steel plate was elevated away from a perforated stainless-steel table using four stainless-steel bolts and nuts (diameter of ½ in.) as shown in Figure 6a,b. The distance between the center of two bolts was set to be 6 in. as seen in Figure 4. Then, the concentrated loads were applied at the center of the steel plate through a simple point load frame as shown in Figure 6a,b. A stainless-steel bar with a diameter of 0.75 in. was welded to a stainless-steel plate with a size of 6 in. × 6 in. × 0.125 in. to provide the point contact between the loads and the coated testing steel plate samples. The cross-section of the stainless-steel bar is large enough to ensure the loads remain stable when the weights are placed on top of the plate during the loading tests. Discrete accumulated weights were placed on top of the stainless-steel plate to generate the static loads. To be statistically valid, three identical specimens were tested in the loading-only tests in addition to a fourth specimen for temperature compensation. The samples for loading-only tests were labeled as L1, L2, L3 for loading tests and LT for temperature compensation.

#### 3.2.2. Determination of the Static Loading Levels

**Figure 7:**
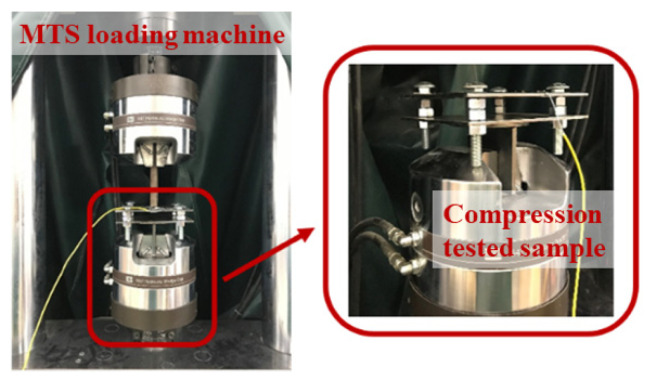
Compression test setup.

To actually determine the loading levels to be applied on the loading frame as shown in Figure 6b for inducing noticeable stress or damages on the epoxy coated steel plate, a compression loading calibration test was conducted to set up a relationship between the strains on the FBG sensor and the static loads on top of the steel plate. Figure 7 shows the test setup. The same test sample as shown in Figure 6 was used to perform the calibration test. Instead of using discrete loads, an MTS loading machine was used to apply a gradually increasing compression load on top of a ¾ in. diameter steel rod. During the calibration test, the same sampling rate of 10 Hz was used for both the FBG interrogator and MTS machine. 

**Figure 8:**
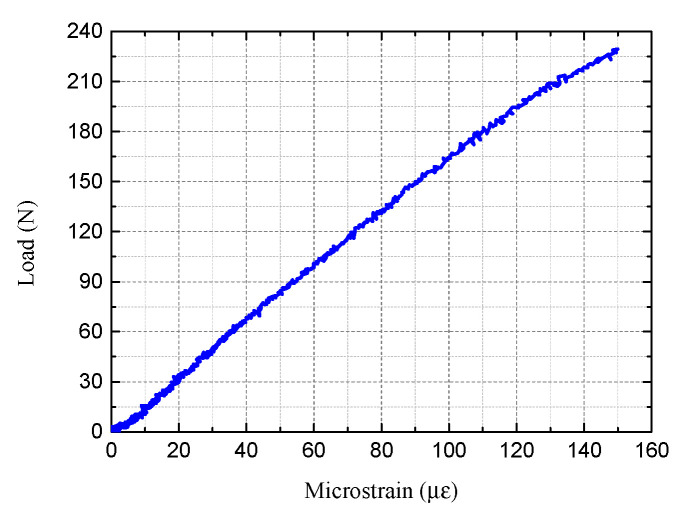
Results from the loading calibration test.

Figure 8 shows the result of compression test. Figure 8 indicated a linear correlation between the obtained strains (µε) from the FBG sensor and the load (N) on top of the plate of 0.6 µε/N.

Since corrosion on epoxy coated steel is a very slow process, corrosion is unlikely to induce sudden shifts of Bragg wavelength for the FBG sensors. On the other hand, loading on the FBG sensors will induce a sudden significant amount of Bragg wavelength shifts. To have noticeable changes in the Bragg wavelength changes induced by loading during the further combined loading–corrosion tests, a 20 pm of Bragg wavelength change induced by a discrete load level was selected to have a more obvious observation. According to Equation (3), a Bragg wavelength shift of 20 pm corresponds to a strain level of approximately 20 µε in the axial direction of the FBG sensor. From Figure 8, it can be seen that 20 µε of strain changes in the axial direction of the FBG sensor required a load increase of 30 N. Therefore, the discrete weight level to be placed on top of the steel plate was selected to be 30 N.

To investigate the impacts of loads on steel corrosion, a five-level loading and unloading test were designed using each load level of 30 N. To produce such a static load level, two steel plates with the size of 6 in. × 6 in. ×6 in. were fabricated and used as weight for 30 N, as shown in Figure 6b. Thus, for the five-level loading and unloading tests, a total of 10 such steel plates were prepared. For each static weight level, a load duration of 2 min was used to ensure a stable load. Table 2 lists the static load values (N) and the corresponding expected strain levels (µε) based on the calibration loading test results in Figure 8 of the five-level loading test.

### 3.3. Corrosion-Only Tests

**Figure 9:**
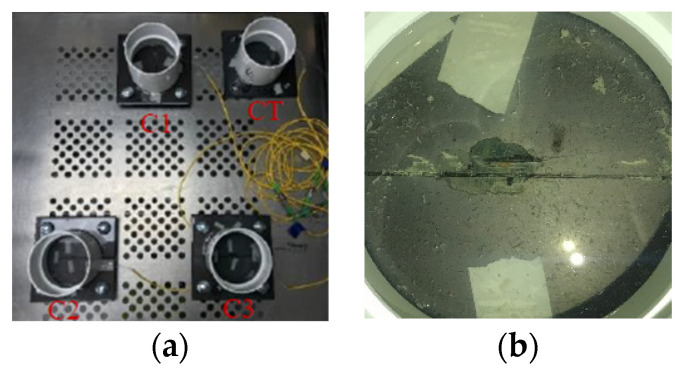
Experimental setup for corrosion-only tests: (**a**) corrosion-only test setup and (**b**) introducing artificial cracks on epoxy coating for accelerated localized corrosion.

Corrosion-only tests were also set up to investigate how the epoxy coated steel behaves under corrosion only environments. To create a corrosion environment on the epoxy coated steel plates, PVC pipes with a diameter of 4 in. were attached to the surfaces of epoxy coated steel plate specimens using epoxy adhesive as shown in Figure 9a. After the adhesive was fully cured, PVC pipes were filled with 3.5 wt% NaCl solution to simulate an accelerate corrosive environment. To be statistically valid, four identical specimens were tested at the same time, one of which was used for temperature compensation without loading and corrosion. The samples for corrosion-only tests were labeled as C1, C2, C3, and CT (CT was used for temperature compensation). Since epoxy coating has great corrosion protection, if there are no defects or damages on the epoxy coatings [48], it would take too long for the corrosion tests if there were no pre-fabricated damages on the epoxy coating. Thus, to accelerate the corrosion process, one artificial crack with a length of 1 in. was introduced to the epoxy coating using a micro-grinder. The artificial crack was located 0.5 in. away from the FBG sensor on each steel plate sample as shown in Figure 9b. In this paper, the corrosion tests were conducted for up to 120 days.

### 3.4. Combined Loading–Corrosion Tests

**Figure 10:**
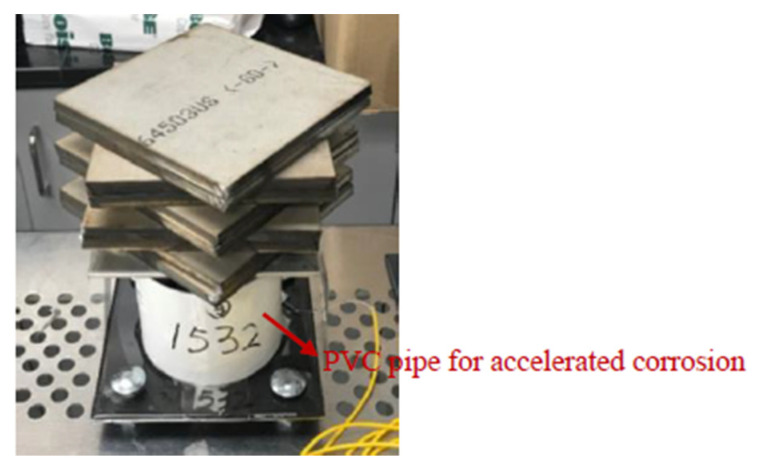
Experimental setup for combined loading–corrosion tests.

To investigate the interaction between load- and corrosion-induced damages, combined loading–corrosion tests were also conducted. Figure 10 shows the designed test setup for the combined loading–corrosion tests. The corrosion test was first set up following the same setup as in Section 3.3. During the corrosion process, there was one cycle of static loading applied to the samples every six days following the loading test setup as in Section 3.2. Each loading cycle lasted 20 min from loading to unloading, and each loading/unloading level lasted 2 min to stabilize the wavelength changes. The combined loading–corrosion tests lasted for 120 days to be consistent with the corrosion only tests. During the 120 days, there were 20 cycles of interactive loading tests performed on each test sample. Table 3 provides the schedule of interactive loading and corrosion tests. The same samples which previously performed loading tests, L1, L2, and L3 were used to conduct the combined loading–corrosion tests renamed B1, B2, and B3. With temperature compensation samples for each test, all the experimental results and analysis in next section are presented after compensating temperature effects.

## 4. Experimental Results

All the following analysis of experimental test results are based on the Bragg wavelength changes induced by loading and corrosion. The corresponding strain changes caused by loading and corrosion in the FBG sensors and the test samples can be calculated according to Equations (3) and (4), which will show the same changing law as the Bragg wavelength changes.

### 4.1. Loading-Only Tests

**Figure 11:**
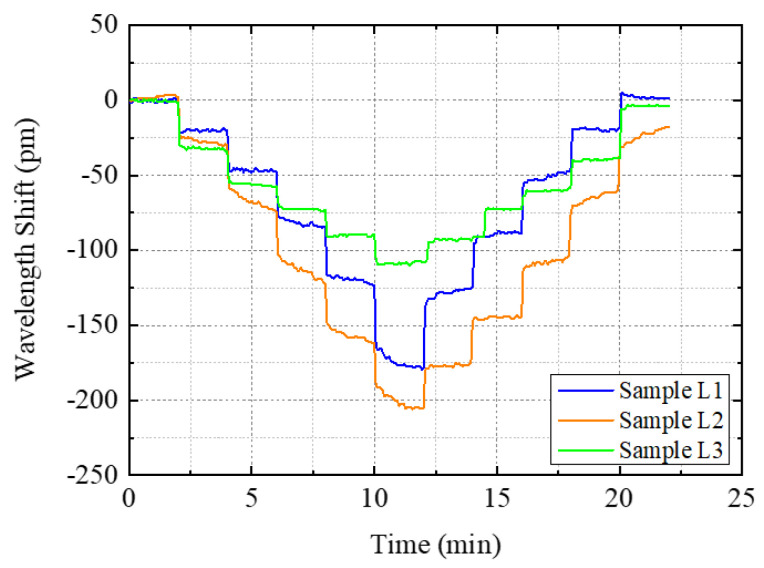
Results of the Bragg wavelength shifts of the samples in the loading and unloading tests.

Figure 11 shows the Bragg wavelength shifts of three testing samples during the five-level loading and unloading tests. As each test sample was tested three times, Figure 11 shows the average wavelength shifts for each sample. From the figure, it is noted that the wavelength changes of all three samples yielded stepwise wavelength changes. The total wavelength shifts of L1, L2, and L3 exceeded 100 pm and reached the expected wavelength variation ranges as expected in Table 1, with 180 pm, 210 pm, and 110 pm, respectively. The variations of differences in strain sensitivity for the three samples accounted for the variance of FBG sensor location during loading and the possibility that a slight bending of FBG sensors existed during samples manufacturing, which revealed the differences in the FBG sensors among the three tested samples. It also provided corresponding comparative control for the analysis of the results of the combined loading–corrosion tests. During the loading interval of 2 min, Sample L1 and Sample L2 showed noticeable increases in wavelength, which may be induced by the inelasticity of epoxy coatings. A longer time interval may be needed for stable loading/unloading of strains. Since this study focuses on the interaction between loading and corrosion, these temporal fluctuations would not impact the analysis results. Figure 11 will be used as control test results to compare with the combined loading–corrosion tests.

### 4.2. Corrosion-Only Tests

**Figure 12:**
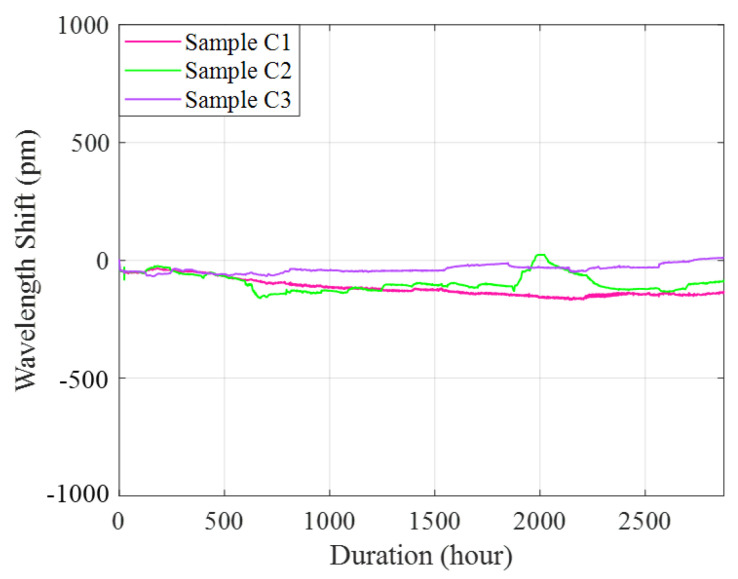
Experimental results from corrosion-only tests.

Figure 12 shows the wavelength changes obtained from the corrosion-only tests. The slight wavelength shift at beginning of the test was induced by the creation of the artificial crack. With the recorded Bragg wavelength changes, the corrosion rate of each sample can be estimated using [26]:

(14) $$CR=\gamma \cdot \frac{d\Delta \lambda }{dt}$$

where, γ is the sensitivity of the sensor to the rate of metal corrosion. Based on Equation (14), Table 4 lists the estimated average corrosion rate during the entire testing period of each sample. The average corrosion rate of the three epoxy-coated steel plate samples with prefabricated crack ranging from 0.28 to 1.13 μm/year.

### 4.3. Combined Loading–Corrosion Tests

**Figure 13:**
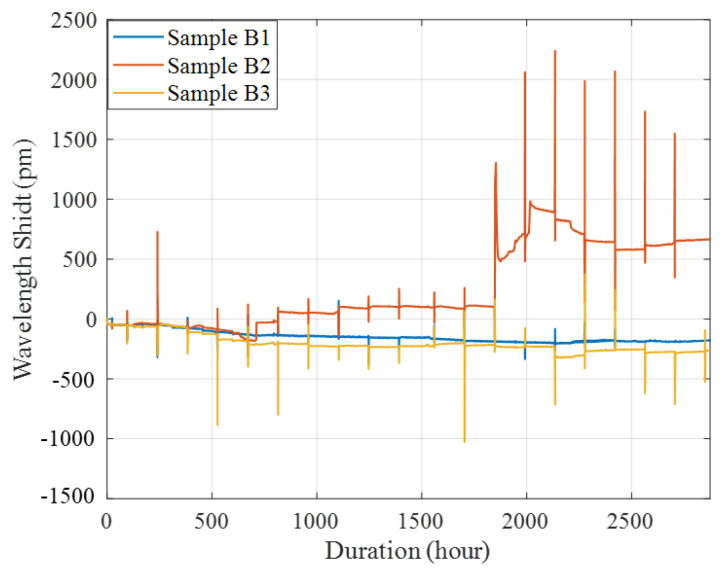
Wavelength shifts of the three tested samples under combined loading–corrosion tests.

**Figure 14:**
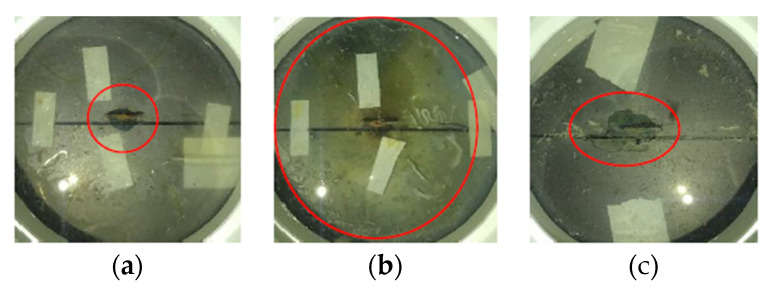
Visual inspections of the three tested samples under combined loading–corrosion tests at the end of the experiments: (**a**) B1, (**b**) B2, and (**c**) B3.

Figure 13 shows the monitored Bragg wavelength shifts of the three tested samples during the entire combined loading–corrosion tests, including the gradual changes caused by corrosion and the sudden changes induced by the loading and unloading cycles. In Figure 13, each sudden wavelength change peak indicates a loading cycle. Due to the long timescale, the change of wavelength for each cycle is shown as a wavelength change peak. Figure 14a–c show the visual inspection of the three tested samples at the end of the combined loading–corrosion tests. From Figure 13, it can be seen that the changing trends of B1 and B3 were basically the same with similar wavelength shifts, indicating that they had experienced a similar extent of corrosion. However, B2 showed a different trend. This different trend of B2 was because the rod holding the weights for static loading was accidental tipped over during the first loading cycle. This accident may have caused premature delamination between the epoxy and the substrate, which may have accelerated the development of corrosion and made the corrosion more serious than B1 and B3, which has been illustrated in Figure 14b, which shows that the epoxy coating of B2 had delaminated, and a large amount of brown-red corrosion products were generated at end of the test. With the premature delamination, a sudden significant amount of wavelength increases was noticed during and after the 6th and the 13th loading cycles, indicating that the loading may have further induced cracks which accelerated corrosion. At around 2000 h, the corrosion rate (CR) of B2 was estimated to be 39.04 µm/year according to Equation (14), which is close to the corrosion rate of 39.26 µm/year for bare A36 structural steel with corrosion cracks from the previous Tafel test [26]. It indicated that the steel under the epoxy coating had been severely corroded with the existences of cracks.

**Figure 15:**
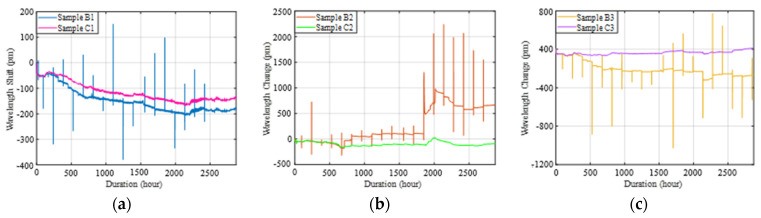
Comparison of recorded Bragg wavelength changes between corrosion-only and combined loading–corrosion tests: (**a**) B1 versus C1, (**b**) B2 versus C2, and (**c**) B3 versus C3.

Figure 15 compares the Bragg wavelength shifts of the three tested samples under corrosion-only and under combined loading–corrosion tests, and Table 5 compares the average corrosion rate during the entire testing period of the corresponding tested samples under these two testing conditions. The results show that external temporary load had promoted corrosion significantly compared to corrosion-only conditions. Specifically, since significant coating delamination occurred for Sample B2 and Sample C2 and Sample B3 and Sample C3, while no coating delamination occurred for Sample B1 and Sample C1, Figure 15 and Table 5 indicate that the effect of loading depended on the condition of the epoxy coating. If delamination exists in the coating, external temporary load can induce severe corrosion up to 12 times higher at the end compared to no external loads, and the interactive damages induced by interaction between corrosion and loads can occur in a relatively short period of time within one week (168 h) after the first loading cycle.

## 5. Discussions

### 5.1. Corrosion Depth Severity Ratio

To investigate the influence of external temporary loading on the corrosion severity, based on the corrosion depth, d, as in Equation (5), in this paper, a corrosion depth severity ratio (*R_d_*) s defined as:

(15) $$R_{d}=d_{0}/d_{1}$$

in which, *d*_0_ is the depth of corrosion with corrosion only and *d*_1_ is the depth of corrosion with combined corrosion with external temporary loading cycles. With an expectation that external loads would increase corrosion depth, the corrosion severity ratio is expected to be smaller than 1 and a smaller corrosion depth severity ratio indicates a higher impact of external temporary loading cycles on the corrosion progressing.

**Figure 16:**
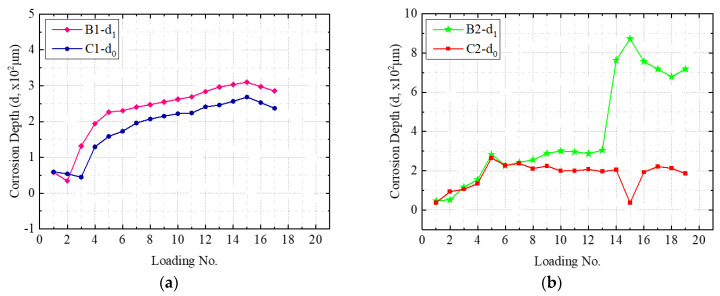

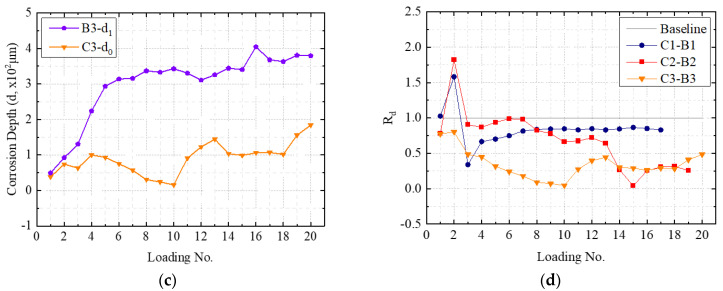
Comparison corrosion depth severity ratio: (**a**) Sample B1-C1, (**b**) Sample B2-C2, (**c**) Sample B3-C3, and (**d**) Corrosion depth severity with impact from external temporary loads.

Figure 16a–c show the estimated corrosion depths, *d*_0_ and *d*_1_, and Figure 16d illustrates the corrosion depth severity ratio, *R_d_*, for all three tested samples. It is noted that for all three samples, the external temporary loading cycles had significantly increased the corrosion depths for all three tested samples. Clear abrupt changes in Figure 16b after Loading No. 5, 14, and 15 indicate that significant and sudden development of pitting corrosion occurred after these loading cycles. Figure 16d clearly indicates that after three loading cycles, all three samples had promoted corrosion severity compared to before three loading cycles. The corrosion depth severity ratio shows a trend to approach zero if coating delamination exists. In addition, since C1–B1 and C2–B2 had showed delamination in the epoxy coating, loading had a more pronounced effect on the corrosion development at the beginning of corrosion.

### 5.2. Corrosion width Severity Ratio

**Figure 17:**
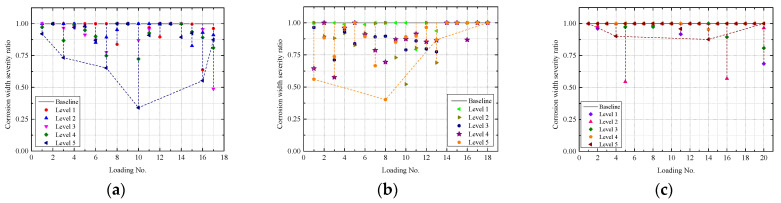
Changes of corrosion width severity ratio ($w_{c}/L_{0}$) under the combined loading–corrosion tests: (**a**) Sample B1, (**b**) Sample B2, and (**c**) Sample B3.

According to Equation (13) and the monitored Bragg wavelength changes in Figure 15 and Figure 17a–c, which show the changes of corrosion width severity ratio, which is defined as dividing the actual corrosion width ($w_{c}$) by the initial assumed corrosion width (*L*_0_), under the combined loading–corrosion tests of the three tested samples with five different loading levels. As the original width of corrosion is unknown, in this study, the original width of the corrosion ($L_{0}$) is assumed to be the diameter of the external load which equals to 0.75 in. (diameter of the loading rod) in this experiment. As the actual corrosion width usually is less than the diameter of the external load (0.75 in.), the corrosion width severity is expected to be a value of less than 1.0. The accumulation of corrosion products under FBG sensors induce an upward lift leading to the increase of the corrosion width, and as the corrosion grows more severe, the corrosion width is expected to be comparable and even larger than the external load diameter. Thus, the corrosion width ratio is expected to increase back to 1.0 or higher as the corrosion continuously grows. From Figs. 17(a–c), among the three samples, the corrosion width ratio of B2 was restored to 1.0, indicating that the corrosion width of B2 grew to be equal to or larger than the diameter of the external load (0.75 in.) at the end of the experiment. In addition, because of the compacting effect of external loads on the corrosion products, most of the contact lengths vary significantly during each loading test from level 1 to level 5. Overall, by comparing the corrosion width ratio of different samples after all the loading tests, it was found that B2 has the largest corrosion width followed by B1 and B3, which is consistent with the findings in Section 4 and Section 5.1.

## 6. Conclusions

Experimental results proved that the FBG sensors were able to accurately monitor corrosion and the significant interactions between corrosion and external loads in real time. The most remarkable findings in this paper are listed below:

1. The wavelength changes among different samples were compared. It was found that delamination of the epoxy coating would significantly accelerate corrosion in epoxy coated steel and increase the contribution of external loads on impacting corrosion.
2. The comparison between the wavelength changes under corrosion-only and combined loading–corrosion tests and the corrosion depth analysis indicated that temporary external loading can significantly accelerate corrosion up to 12 times compared to no external loading, especially in the condition when the coating was delaminated.
3. With combined external temporary loading and corrosion, the variation of restoration of corrosion width ratio went back to a value closer to 1.0, indicating the influence of the compacting effect of loose corrosion products under external loading.

The current studies were conducted in laboratory environments which may limit the effects of loading on corrosion. In the future, further study will be performed to assess the long-term interaction of corrosion and static loads in practical applications.

## Figures and Tables

**Table 1:** Properties of the FBG sensors.

FBG Length	10 mm
Strain limit	5000 με
Strain sensitivity	~1.2 pm/με
Operating temperature range	−40 to 120 ℃
Thermal sensitivity	~9.9 pm/°C
Fiber coating	Polyimide
Fiber re-coating diameter	145–165 μm

**Table 2:** Load values and corresponding expected strain levels of the five-level loading test.

Load Level	Load Value (N)	Expected Strain Value (µε)
1st	30	18
2nd	60	36
3rd	90	54
4th	120	72
5th	150	90

**Table 3:** Schedule of interactive loading and corrosion tests.

Time (Days)	Test Operation
0	Corrosion test start
4	1st cycle of loading test
10	2nd cycle of loading test
16	3rd cycle of loading test
22	4th cycle loading test
…	…
120	20th cycle of loading test
120	Corrosion test end

**Table 4:** Average corrosion rates of three samples.

Sample No.	Corrosion Rate for Corrosion-Only Conditions (µm/year)
C1	1.13
C2	0.52
C3	0.28

**Table 5:** Measured average corrosion rates of the tested samples.

Samples	Corrosion-Only Average Corrosion Rate (µm/year)	Combined Loading–Corrosion Average Corrosion Rate (µm/year)	Percentage of Corrosion Rate Increase (%)
1	1.13	1.63	44.3%
2	0.52	6.80	1207.7%
3	0.28	1.72	514.3%

## Data Availability

Not applicable.
